# Copper Deficiency Myeloneuropathy in Autoimmune Disease

**DOI:** 10.7759/cureus.16591

**Published:** 2021-07-23

**Authors:** Jonathan T Grossman, Steven Ruiz

**Affiliations:** 1 Neurology, Florida Atlantic University Charles E. Schmidt College of Medicine, Boca Raton, USA; 2 Internal Medicine, Florida Atlantic University Charles E. Schmidt College of Medicine, Boca Raton, USA

**Keywords:** copper deficiency, copper-induced myelopathy, myeloneuropathy, diffuse systemic sclerosis, auto-immune, subacute combined degeneration, tabes dorsalis, serum vitamin b12

## Abstract

Copper deficiency is a rare and potentially treatable cause of myeloneuropathy. The most common causes of acquired copper deficiency include malabsorption following gastric surgery and excessive zinc supplementation. Clinical manifestations can be localized to the dorsal spinal cord and present similarly to those that characterize classic vitamin B12 deficiency. In this report, we present the case of a 76-year-old female with copper deficiency myeloneuropathy as a presumed consequence of advanced systemic sclerosis (SSc).

## Introduction

Copper deficiency myeloneuropathy, acquired or otherwise, is a rare condition. While known to cause anemia and leukopenia, the implications of copper deficiency in myelopathy have only been recently unearthed, having just been discovered near the end of the 20th century [1]. Historically, a majority of the cases of copper deficiency were mistakenly diagnosed as subacute combined degeneration of the spinal cord. This is because its hallmarks - spastic gait and sensory ataxia - resemble those of vitamin B12 deficiency. While the pathophysiology underlying the myelopathic features of copper deficiency remains unclear, copper is known to play a key role in the function of many enzymes with essential roles in maintaining and regulating cellular health and function. It has been postulated that copper may play an integral role in methylation via methionine synthase, a necessary step in the production of purines and myelin proteins [2]. Copper is also a cofactor for the enzyme superoxide dismutase, which is involved in the scavenging of free radicals. Additionally, it is a known cofactor in cytochrome C oxidase-driven electron transport and oxidative phosphorylation, both crucial processes in the production of adenosine triphosphate (ATP). It remains unclear whether deficiencies in a combination of the above processes are responsible for the damage that precipitates myelopathy.

The most common causes of acquired copper deficiency include malabsorption as a consequence of prior gastric surgery or enteropathy (e.g., celiac disease, cystic fibrosis, and inflammatory bowel disease) and excessive zinc supplementation [3,4]. Given that most dietary copper is absorbed in the duodenum, the potential deleterious effect of gastric surgery on absorption is obvious. Further, zinc and copper compete for absorption across the intestinal brush border through the binding of an intracellular protein called metallothionein. Consequently, excessive zinc supplementation can impair the absorption of copper into enterohepatic circulation.

There is scarce literature describing patients with acquired copper deficiency as a consequence of gastrointestinal (GI) dysfunction in autoimmune disease. In light of this, this case report discusses a 76-year-old female with copper deficiency myeloneuropathy as a presumed consequence of advanced systemic sclerosis (SSc).

## Case presentation

A 76-year-old female with a past medical history significant for esophageal dysmotility secondary to Sjogren's syndrome (SS) and systemic lupus erythematosus presented for the evaluation of subacute, progressive upper and lower extremity weakness and incoordination that had begun two months prior. The onset of these symptoms had been preceded by lower back pain. The patient had no history of gait difficulties. From the onset of her deficits, she had experienced a precipitous decline; she found it difficult to hold things in her hand for a prolonged period of time, her writing had become illegible, and she could no longer ambulate without the assistance of a rolling walker. She reported a sense of "stiffness" in her legs that she noticed only when ambulating. She also described numbness in her hands and feet.

Her BMI upon admission was 14 kg/m^2^, which she reported was a direct consequence of chronically decreased oral intake due to dysphagia. Her daily caloric intake was often limited to a single meal consisting of either a cup of soup or a single pureed meal. She admitted that her diet was near-devoid of any form of animal protein. While she had struggled with dysphagia for many years, she had never experienced a constellation of symptoms such as this in the past.

Preliminary labs included a complete blood count, comprehensive metabolic panel, magnesium, phosphorus, and creatine kinase. Of these, remarkable values were found for red cell count (RCC): 3.38 x 10^6^ mcL (normal range: 4.2-5.4), hemoglobin: 10.6 g/dL (12-16), hematocrit: 35.4% (37-47), and mean corpuscular volume (MCV): 104.7 fL (81-99). She underwent imaging including CT lumbar spine and MRI cervical/thoracic and lumbar spine without contrast. These imaging studies were ordered prior to consulting our inpatient Neurology service.

**Figure 1:**
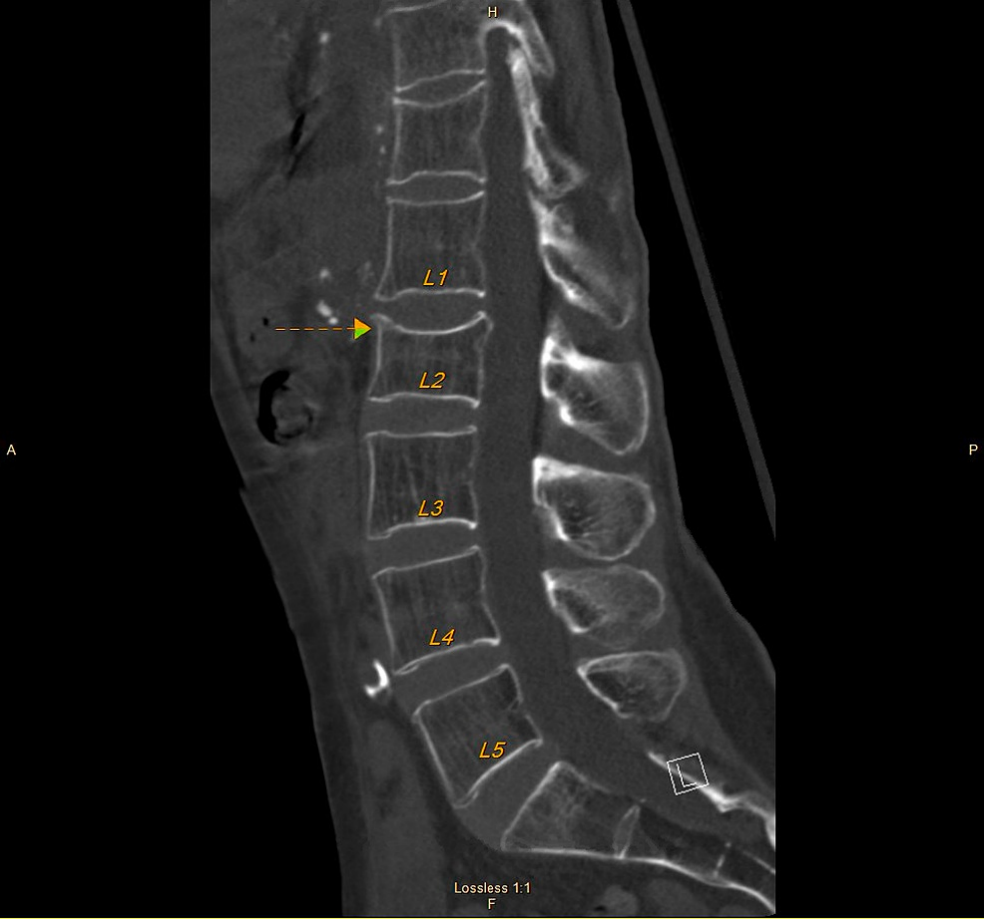
NCCT image of the lumbar spine: sagittal view The image shows an incidental finding of anterior step defect with a vague line of condensation just inferior to this consistent with a subacute L2 compression fracture NCCT: non-contrast computed tomography; L2: second lumbar vertebra

The CT lumbar spine showed an incidental finding of subacute L2 compression fracture (Figure 1).

**Figure 2:**
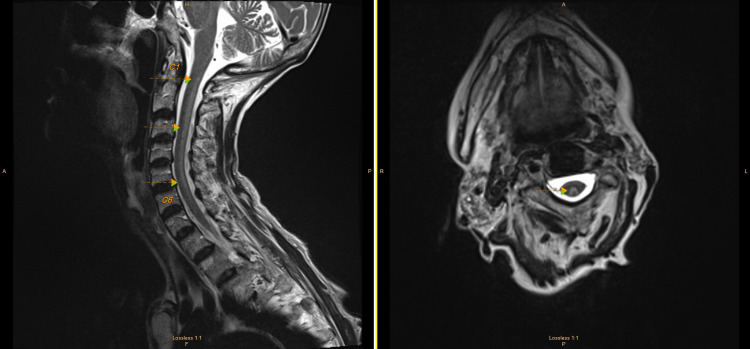
MRI cervical spine Sagittal T2WI (left) shows increased signal in the dorsal aspect of the spinal cord spanning C1-C6 with corresponding signal change on axial T2WI (right), often described as an inverted "v." T2WI: T2-weighted imaging; C1: first cervical vertebra; C6: sixth cervical vertebra

MRI cervical spine showed increased signal intensity throughout the dorsal column spanning C1-C6 (Figure 2).

The initial physical examination performed by our service was remarkable for decreased grip strength and 3/5 hip flexion bilaterally. There was also impaired proprioception in all four extremities, decreased vibratory sensation limited to the lower extremities, and positive Romberg. Notable negative findings included the absence of afferent pupillary defect and hyperesthesia, normal tone and heel-shin test, and symmetric deep tendon reflexes throughout. This exam in combination with MRI findings suggested likely dorsal column disease. Given her history, suspicion was high for underlying vitamin or trace mineral deficiency. Tabes dorsalis, of course, was also a consideration. The lumbar puncture was unrevealing. Additional labs were ordered, including rapid plasma reagin (RPR) (nonreactive) as well as serum vitamin B1, B3, B6, B12, and folate, which were all within the reference range. Serum zinc was 46.6 mcg/dL, just below the lower limit of normal (60-120). Serum copper was markedly reduced at 33.8 mcg/dL (80-155). Neither 24-hour urine zinc nor serum ceruloplasmin was measured.

Considering the temporal evolution of these symptoms, daily IV copper infusion (2 mg/day) was initiated and continued for a total of five days. The patient experienced interval improvement in strength, sensation, and balance during her stay. She was eventually able to ambulate without the assistance of her rolling walker, which she had been dependent on for nearly two months. Nutrition services provided guidance regarding maintenance of adequate daily oral intake of copper including a designated discharge meal plan. Physical examination on the day of discharge was remarkable for near-complete resolution of deficits in proprioception and vibratory sensation. She was discharged with instruction to follow up with nutrition services as well as our outpatient Neurology Residency Clinic in two weeks for re-evaluation including a repeat MRI C-spine.

## Discussion

The mean daily dietary intake of copper in America is estimated at 800-1000 mcg for children and 1100-1400 mcg for adults, with a recommended daily dietary allowance (RDA) of 340-890 mcg and 890-1300 mcg, respectively [5,6]. Thus, the typical American diet meets or exceeds the recommended dietary allowance of copper.

It can be reliably speculated that a combination of factors contributed to the evolution of deficiency in our patient: (a) dysphagia resulting in chronically decreased oral intake, (b) gastroparesis, and (c) antacid-mediated changes in intraluminal pH. Further, other trace minerals can compete with copper for absorption. Iron and zinc are two well-studied vital minerals that can affect copper absorption. Iron ingested in relatively large amounts decreases copper absorption in both rats and humans [7]. Daily zinc intake in amounts as low as 18.5 mg (but typically ~40 mg or more) has been associated with impaired absorption and diminished copper status [8]. Our patient was not using, nor had she ever used, iron or zinc supplements prior to the presentation. Other common factors that may influence copper status include the total amount the body stores (which influences the rate of intestinal copper absorption) and vitamin C supplementation (via reduction of copper to the less absorbable cuprous state) [9].

GI dysfunction is known to occur with many autoimmune diseases. While our patient had carried a diagnosis of SS from the remote past, she lacked the classic ocular or oral manifestations of exocrine gland dysfunction. She also denied having undergone any confirmatory serologic testing. Further, other findings including epidermal thickening of the face and hands, fluctuating digital pain, dyspnea on exertion (in the absence of underlying cardiovascular pathology), and intermittent nonproductive cough were symptoms that appeared more consistent with SSc. Esophageal dysmotility leading to dysphagia has been observed in SSc [10]. While we were suspicious that she may have been misdiagnosed with SS, both SS and SSc can lead to similar nutritional deficiencies. Therefore, we determined that, for our purposes, the distinction was unnecessary.

The term scleroderma directly translates to "thick skin." SSc, a form of scleroderma, is a diffuse, debilitating disease caused by inappropriate activation of fibroblasts driven by vascular and immunological factors. The result is abnormal collagen deposition in both the skin and internal organs. Approximately 90-95% of patients with SSc will experience some form of GI involvement, the leading cause of morbidity and the third most common cause of mortality [11]. GI involvement has been observed in both limited and diffuse cutaneous forms of the disease. The esophagus is typically the initial and most commonly affected section of the GI tract. Esophageal dysfunction occurs due to atrophy of the smooth muscle of the lower two-thirds of the esophagus including the lower esophageal sphincter. This leads to weak peristaltic movements, reflux, and an increased risk of esophageal strictures. The associated discomfort and dysphagia often lead to chronically decreased oral intake and subsequent malnourishment. Decreased caloric intake and limited food options lead to a dietary deficiency in many trace minerals including copper.

Gastroparesis has also been observed in approximately 38-50% of patients with systemic scleroderma [12,13]. This may contribute to malabsorption via a combination of factors including food aversion and early satiety, bloating, nausea, and vomiting as a consequence of delayed gastric emptying. While our patient experienced symptoms consistent with gastroparesis, she had never undergone a gastric-emptying study nor had she been diagnosed with gastroparesis in the past, and thus had not incorporated dietary changes aimed at improving gastric emptying, i.e., reduced fat and fiber intake. An association has been demonstrated between gastroparesis and mineral deficiencies including iron and zinc [14]. While iron studies were not performed during her hospitalization, our patient presented with decreased RCC, hemoglobin, and hematocrit with elevated MCV; B12 and folate were within the reference range. Because iron requires ceruloplasmin for oxidation and binding to transferrin (and total body stores of copper directly influence ceruloplasmin production), low serum copper levels are typically associated with microcytic anemia due to iron deficiency. This is a direct consequence of impaired transport of iron. As anticipated, her serum zinc level was below the lower limit of normal: 46.6 mcg/dL (60-120).

Finally, small intestinal bowel overgrowth (SIBO), a disorder characterized by symptoms of abdominal pain, early satiety, diarrhea, bloating, and flatulence, has been observed in many autoimmune diseases including both SS and SSc [15,16]. While data is limited regarding the prevalence of SIBO in SSc, a recent systematic review reported a prevalence ranging from 12-55% [16]. The exact underlying pathophysiology remains unknown. It can lead to similar nutritional deficiencies as observed in malabsorption syndromes including fat-soluble vitamins, vitamin B12, iron, and folate [17].

## Conclusions

This case report highlights the importance of considering a less common trace mineral deficiency in patients who present for the evaluation of subacute gait dysfunction without a history of gastric surgery or excessive vitamin/trace mineral supplementation. While we questioned the reliability of our patient's outpatient diagnoses of SS and systemic lupus erythematosus (especially in the absence of classical clinical signs/symptoms and confirmatory serologic testing), we considered a distinction between or among these disorders and SSc to be unnecessary as GI dysfunction has been described in all of these conditions. While the exact pathophysiology underlying central nervous system dysfunction in copper deficiency remains uncertain, interval recovery is possible with expedient parenteral and continued enteral supplementation. If overlooked, acquired copper deficiency as a consequence of autoimmune disease-associated GI dysfunction can lead to significant morbidity and mortality.
